# Self-Perceived Stress and the Personality of Mothers of Children with Central Auditory Processing Disorder, as Well as in Mothers of Typically Developing Children, Before and Late in the COVID-19 Pandemic

**DOI:** 10.3390/audiolres14050076

**Published:** 2024-10-15

**Authors:** Joanna Kobosko, Lech Śliwa, Malgorzata Ganc, W. Wiktor Jedrzejczak, Henryk Skarzynski

**Affiliations:** 1 Institute of Physiology and Pathology of Hearing, Mochnackiego 10, 02-042 Warsaw, Poland; j.kobosko@ifps.org.pl (J.K.); l.sliwa@ifps.org.pl (L.Ś.); m.ganc@ifps.org.pl (M.G.); skarzynski.henryk@ifps.org.pl (H.S.); 2 World Hearing Center, Mokra 17, Kajetany, 05-830 Nadarzyn, Poland

**Keywords:** stress, Big Five personality traits, auditory processing disorder, COVID-19

## Abstract

Objectives: This study aims to measure, at two time points, the relationship between self-perceived global stress and the personality traits of the mothers of children who have central auditory processing disorder (APD) and compare it with the results from mothers of typically developing (TD) children. The comparisons were made before the COVID-19 pandemic, as well as late in the pandemic. Methods: The level of stress was assessed using the Perceived Stress Scale (PSS-10), while the Short Big Five Markers (IPIP-BFM-20) were used to assess Big Five personality traits. The study used two independent samples: one evaluated before the COVID-19 pandemic and the other late in the pandemic. Each sample consisted of 108 mothers of children with APD and 79 mothers whose children did not have APD (TD children) as controls. Results: The average global stress levels were similar in mothers of children with APD and in mothers of TD children, both before and in the late stage of the COVID-19 pandemic. During the late stage, both sets of mothers scored similarly on all personality dimensions, but significantly, mothers of TD children exhibited lower emotional stability compared to during the pre-pandemic period. In both groups, emotional stability predicted global stress level at both time points; however, during the pandemic, conscientiousness also became a predictor of global stress level but only in the group of mothers of TD children. Conclusions: Mothers of APD children might be more resistant to additional stressors. It would be prudent to watch mothers of APD and TD children for signs of needing psychological intervention.

## 1. Introduction

The COVID-19 pandemic, especially in its early phase from March to May 2020, led to numerous restrictions, including lockdowns, social distancing, the closing of universities, schools, kindergartens, and playgrounds, remote work and education, quarantines, and remote medical and rehabilitation care. Unfamiliarity and uncertainty about the disease gave parents new and unfamiliar stressors [1,2]. The pandemic raised general stress, defined as the tension between a person and their environment, such that the person feels there is an extra burden that might threaten their well-being and exceed their resources [3]. Nonetheless, individuals will react differently depending on their personality traits and other factors.

Soon after the outbreak of COVID-19, most adults around the world experienced, on average, moderate global stress [4]. This included parents who were confronted during that time with multiple extra parental tasks [1,5]. As many studies have confirmed, the impact of the pandemic involved an increase in self-perceived parenting stress [5,6]. Increased stress levels due to COVID-19 were associated with increased depression, anxiety, and higher parental fatigue [5,7], particularly among those who were at increased risk because of their child’s disability [8] or ill health [9].

In the early stage of the COVID-19 pandemic, some studies also looked at how parents’ personality traits were related to stress and distress [10,11]. The personality traits in these studies made use of the ‘Big Five’ model, which includes extraversion, agreeableness, conscientiousness, openness, and neuroticism (with emotional stability being at the extreme end of the latter) [12]. Results showed that emotional stability was a major factor in determining mental health during the pandemic [10].

Neuroticism (low emotional stability) is related to a tendency to perceive the surrounding world as threatening and psychologically distressing; people suffering from the condition are prone to negative emotional states such as irritability, anger, sadness, anxiety, worry, hostility, and vulnerability [12]. Focusing on parents, studies have confirmed that higher neuroticism plays a significant role in elevating perceived distress, while lower extraversion decreases it [10]. According to studies done before the pandemic, a child’s disability or ill health modifies the relationship between personality traits and global or parental stress [13,14]. Here, emotional stability plays a leading role and proves to be a significant predictor of global stress, such as in mothers of children with auditory processing disorder [13]. Another study on a group of parents of children with autism or intellectual disability reported similar results in terms of neuroticism and parental stress during the early stage of the COVID-19 pandemic; it also found that agreeableness is a protective factor against stress [8].

Auditory processing disorder (APD) in children is a difficulty in processing auditory information in the central nervous system. The British Society of Audiology states that the disorder is caused by abnormal brain function characterized by incorrect differentiation, separation, grouping, localization, and ordering of stimuli. APD manifests as difficulties in understanding speech, especially in challenging listening conditions, and may contribute to problems in language development and school difficulties [15]. According to the American Speech-Language-Hearing Association [16], people with APD have deficits in higher auditory functions, including sound localization, differentiation, recognition of sound patterns, and the ability to understand distorted speech or speech in the presence of noise.

APD may be diagnosed in children of normal intelligence and hearing [17]. It occurs in 0.2–5% of the pediatric population [18], seldom in isolated form, and more often in conjunction with other neurodevelopmental problems [19]. Of children aged 7–12 with APD, some 47% have abnormal language development and reading difficulties [20], and about 25% are diagnosed with developmental dyslexia [21]. It has also been found that APD often coincides with attention deficit hyperactivity disorder or with autism spectrum disorder [22]. Children with APD show difficulties not only in auditory perception, speech, and communication but also in motor and socio-emotional development [23,24].

This work aimed to study the relationship between self-perceived global stress and the Big Five personality traits in mothers of children with APD and in typically developing (TD) children during the late stage of the COVID-19 pandemic (i.e., July 2021 to September 2022) and compare these results with those from an independent group of mothers obtained before the pandemic. Another aim of the study was to examine differences between mothers of children with APD and those with TD in terms of levels of self-perceived global stress and Big Five personality traits, and to relate them to existing Polish norms for the general population. Finally, we investigated whether there were differential effects of having a child with APD (vs. TD) on mothers’ self-perceived global stress and Big Five personality traits, and whether the effects differed between pre-pandemic vs. late pandemic.

## 2. Materials and Methods

### 2.1. Study Design and Participants

This work replicates and builds on a study conducted before the COVID-19 pandemic [13] and was carried out late in the COVID-19 pandemic period, from July 2021 to September 2022. It had an anonymous, cross-sectional research design and was applied to mothers of children with APD as well as mothers of TD children, all of whom were residents of central Poland. The groups were independent of those assembled for the previous study conducted before the COVID-19 pandemic [13]. The mothers of the APD children completed questionnaires during a 5-day hospital stay organized for the children by the Institute of Physiology and Pathology of Hearing. During that time, the children underwent auditory therapy involving auditory training with active and passive learning. Participation in the study was voluntary; the response rate was 50%.

The APD children were diagnosed with the condition by an audiologist based on the ASHA criteria (2005). The children were given a battery of psychoacoustic tests to gauge central auditory function: the Dichotic Digit Test (DDT), the Frequency Pattern Test (FPT), and the Duration Pattern Test (DPT) (e.g., [25]). An exclusion criterion was the coexistence of APD with other disorders such as ADHD, dyslexia, specific language impairment, attention deficit disorder, autism spectrum disorder, developmental delay, or other neuropsychiatric or somatic disorder.

Mothers of TD children were recruited by teachers in primary and secondary schools, again on a voluntary basis. Most mothers had received higher education. According to information provided by the mothers in the survey, all TD children were healthy and did not manifest developmental difficulties at the time of the study. After completing questionnaires, mothers placed them in sealed envelopes and in special boxes set up at the schools. Table 1 lists the sociodemographic data on mothers and their children. Data collected in the present study will be compared to data collected earlier on a different group of subjects (Table 1 of [13]).

### 2.2. Measures

Self-perceived global stress was measured using the Perceived Stress Scale (PSS-10; [26]) in its Polish adaptation [27]. PSS-10 is a self-report tool designed to subjectively measure perceived stress levels. It contains 10 questions about potentially stressful situations encountered over the past month and the ways used to cope with them. Replies are marked on a Likert-type scale from 0 (never) to 4 (very often). The total score in the range 0–40 represents the global stress score, with a higher score indicating higher global stress. Cronbach’s alpha was 0.86 in the group of mothers of APD children and 0.89 in mothers of TD children.

Personality traits in the Big Five model were assessed using the Polish adaptation of the Short Form IPIP-BFM-20 questionnaire [28]. It contains 20 items, four for each subscale spanning one of the Big Five personality traits: extraversion, agreeableness, conscientiousness, emotional stability, and intellect/imagination. The subject uses a Likert-type scale to register how accurately each item describes them (from 1, strongly disagree, to 5, strongly agree). The higher the score on a given scale (ranging from 4 to 20), the stronger this personality trait. Cronbach’s alpha in the group of mothers with APD children ranged from 0.76 for extraversion to 0.5 for intellect/imagination; in the group of mothers of TD children, it ranged from 0.81 for extraversion to 0.5 for intellect/imagination.

### 2.3. Statistical Analysis

Statistical analysis was performed using Statistica v. 12 (StatSoft Inc., Tulsa, OK, USA). The following tests were applied: Pearson χ^2^ (for assessing differences between sociodemographic data), two-factor ANOVA (for comparisons of all groups of subjects), Student’s t-test (for assessing differences between mothers of TD or APD children before and late in the COVID-19 pandemic), Pearson correlation coefficient (for correlating stress level and personality dimensions), and multiple linear regression (for assessing which variables predicted the stress level of mothers). A confidence level of 95% (*p* < 0.05) was chosen as the criterion of significance. In ANOVA, we verified normality of the variables (Shapiro–Wilk test) and the assumption of homogeneity of variances (Levene’s test). According to test results, the assumptions were not violated in any case. In multiple regressions, we tested normality of residuals (Shapiro–Wilk test). Additionally, we applied the Durbin–Watson *D*-statistics to check for serial correlation between residuals. The tests confirmed normality and independence of variables.

## 3. Results

We assembled exactly the same number of subjects as in [13], but the study period covered the late stage of the COVID-19 pandemic. We tried to collect data matched by age and gender. Table 1 presents the sociodemographic factors related to mothers of children with APD as well as mothers of TD children (the control group). The APD and TD groups differed significantly only in the education level of the mothers (similar to the study conducted before the COVID-19 pandemic) and in the education level of the children, although there was no statistically significant difference in terms of age.

ANOVA was used to test the effects of subject group (mothers of APD children vs. mothers of TD children), COVID-19 pandemic stage (before vs. late), and the interaction of group and COVID-19 pandemic stage (Table 2). The levels of self-perceived stress (PSS-10) and personality characteristics (IPIP-BFM-20) in mothers of APD children and mothers of TD children during the late COVID-19 stage are shown in Table 3 and Table 4, together with comparable data from before the pandemic (taken from Table 2 of [13]). ANOVA showed some significant differences between mothers of APD children and mothers of TD children in terms of personality characteristics (agreeableness and intellect/imagination) regardless of COVID-19 stage (second column of Table 2). There was no main effect of COVID-19 stage on stress level or personality characteristics (third column of Table 2) except for a significant interaction between group and COVID-19 stage for emotional stability. This effect was further investigated with pairwise comparisons for each group. Table 3 shows comparisons between the self-perceived stress level and personality characteristics before and during the late stage of COVID-19 in mothers of children with APD. There were no significant differences. However, a similar comparison made on the control group of mothers of TD children showed a significant decrease in their emotional stability (Table 4).

Next, we examined correlations between the self-perceived level of stress in mothers of APD children and their personality characteristics during the late period of the COVID-19 pandemic (Table 5). Here we see that stress correlated moderately with emotional stability and, to a lesser degree, with extraversion, agreeableness, and intellect/imagination. All correlations were negative. When a similar analysis was done on the group of mothers of TD children, the stress level correlated significantly only with emotional stability and extraversion (Table 5).

Finally, we conducted regression analysis regarding the self-perceived stress level of mothers of APD children and mothers of TD children during the late period of the COVID-19 pandemic (Table 6). The stress level was taken to be the dependent variable, and personality dimensions were taken as descriptors. For mothers of APD children the only significant predictor was emotional stability. However, in the group of mothers of TD children, while emotional stability was still a significant predictor of self-perceived stress level, there was also another significant predictor: conscientiousness.

## 4. Discussion

In the presented study, we investigated relationships between changes in self-perceived stress and the Big Five personality traits in mothers of children with APD and in mothers of TD children, both before the COVID-19 pandemic and during its later phase (1.3 to 2.5 years from its onset). We also explored whether there was an effect of the timing of the study (pre-COVID-19 vs. late COVID-19)—and the presence of APD vs. typical development in children (APD vs. TD)—on the level of self-perceived stress and the Big Five personality traits in mothers. 

The global stress levels experienced by mothers of both APD and TD children were similar, whether before the COVID-19 pandemic or in the late pandemic period, and corresponded to the average global stress level in the general population (according to pre-pandemic norms). Noting reports of a significantly higher level of global stress during the early COVID-19 pandemic in the general population (e.g., [4]) as well as in parents [5], we can presume that at the time our study was conducted, the intensity of global stress in mothers had already returned to a pre-pandemic level. Here, it should be noted that we studied global stress in mothers, not parenting stress, which may still have been elevated in selected aspects [29], even during the late COVID-19 pandemic period. 

However, it is a significant result that all the Big Five personality traits in mothers of APD children were at lower levels than the average for the Polish population [30], both before and during the pandemic. Such a result suggests worse psychological well-being in these mothers [30] and possibly higher levels of parenting stress and distress, aspects that were not directly investigated in this study. 

We found that three personality traits mothers of TD children were significantly lower than pre-pandemic norms: emotional stability, conscientiousness, and intellect/imagination. Lower levels of these personality traits are consistent with a general downward trend observed among the adult American population after the onset of the COVID-19 pandemic [31]. Interestingly, a longitudinal study of the general German population conducted between December 2019 and December 2022 found that there were increases (but not decreases) in extraversion, conscientiousness, and emotional stability during this time, and a decrease only in openness [32], suggesting there might be cross-cultural differences.

Our results reveal decreased well-being in mothers of TD children and, to a greater degree, in mothers of APD children. This result is in line with a study of parents from the German general population over a two-year period of the COVID-19 pandemic showing that they had worse mental health outcomes after almost two years of the pandemic than they did at the beginning [33]. With a deterioration in parental psychological functioning, the risk of child maltreatment increases, which in turn is associated with a higher occurrence of emotional and behavioral difficulties in children, both in those with preexisting health/psychological problems and in typically developing children [6,8,10,33].

The main effect of the child disorder factor (APD vs. TD) observed in the study indicates that it affected two personality traits—agreeableness and intellect/imagination—independently of the other factor, pandemic phase (pre- vs. late COVID-19). That outcome shows that mothers of APD children had fewer psychological resources (in terms of their personality traits) to cope with stressors during the late pandemic. This result is supported by other studies that show that agreeableness plays a key role in complying with recommendations and restrictions [34]. Moreover, studies on parents of children with special needs indicate that, during the pandemic, agreeableness and neuroticism both moderate the effect of parenting stress brought on by the child’s special needs [8]. This implies that a friendly and empathetic mother, focused on pro-social and cooperative behavior with her child (and alsoless neurotic), tends to experience lower levels of parenting stress when having to deal with their child’s special needs (such as those associated with APD). Openness to experience (measured by intellect/imagination, meaning being more open, creative, or imaginative with the child) has been associated with resistance to pandemic stress [35], and, presumably, might mean being better able to cope with challenging behavior in the child, either APD or TD.

Furthermore, the results show that in the compared periods, only mothers with TD children showed a change in the level of one of the personality traits, i.e., their emotional stability decreased. Our study confirms the crucial role that emotional stability plays, in mothers from both groups, in lowering the intensity of global stress, a result similar to findings from other studies on parents (e.g., [8,14]). In the late pandemic period, behaviors related to conscientiousness – such as being organized, diligent, dutiful, thorough, efficient, and systematic – started to have a stronger effect in mothers with TD children [12]. Other studies have shown that conscientiousness is conducive to better health and well-being [36] and to undertaking preventive behaviors during the COVID-19 pandemic [37]. Conscientiousness is also related to lower levels of depression and anxiety, better health, and lower COVID-19-related anxiety [38]. In mothers with APD children, a correlation between perceived global stress and conscientiousness was not observed in the late pandemic period, although it had been present before the pandemic; this indicates that the role of conscientiousness changed in that group of mothers when they were confronted with stresses arising from the earlier stages of the pandemic. 

Comparing results before and during the pandemic, extraversion remained a significant personality trait from the perspective of perceived global stress in both groups of mothers. In the first place, extraversion is related to ease in establishing social relationships, the level of activity and energy, and assertiveness. These behavioral characteristics were important during the COVID-19 pandemic, affecting mother–child interactions. 

We observed a significant interaction between the factor of child disorder (APD vs. TD) and the factor of epidemic phase (pre-COVID-19 vs. late COVID-19) when the trait of emotional stability was considered. These results may be explained by the effects of experiences encountered during the early stage of the pandemic. A correlation between these experiences and psychological functioning, including global stress and personality traits, has already been documented, including in parents (e.g., [6,9,31]).

The hypothesis can be made that because of this interaction, the COVID-19 pandemic did not cause any significant decrease in the emotional stability of mothers with APD children, in contrast to mothers with TD children who had significantly lower emotional stability than before the pandemic—in fact, at a level similar to those mothers with APD children, i.e., with accordingly higher levels of anxiety, depression, distress, mood swings, proneness to worry, hypersensitivity, anger, and annoyance [12]. Other factors that were likely present in the group of mothers of TD children during the COVID-19 pandemic and were not controlled for in this study (such as those related to marital conflicts or loss of loved ones infected with coronavirus) may be responsible for this outcome.

Mothers with APD children, whose emotional stability was comparatively lower before the pandemic, did not experience a further decrease in that trait in the late COVID-19 period—one of the possible reasons being that COVID-19, as well as bringing difficulties and restrictions, might also have brought reduced activity and respite from daily challenges and struggles that confront APD children and their mothers daily, including school, APD difficulties, socio-emotional problems, and peer interactions [23,24]. Moreover, mothers with APD children might also have experienced positive aspects of parenthood during the pandemic: increased emotional closeness and more time for pleasurable joint activities with their children, which could counteract COVID-19 stresses [2]. These positives could serve as a protective factor and maintain emotional stability, which, in this group of mothers, was on the same level as before the pandemic.

A limitation of this study was the use of separately recruited groups to compare the results before and during the COVID-19 pandemic, and this might have affected the results. The anonymity of participation in the pre-pandemic study prevented their repeated participation in the later study, and consequently made longitudinal studies impossible. Another limitation is that the subjects studied were all residents of central Poland. It is also important to consider the possibility of bias in this study, as the recruitment of participants is not broadly targeted to all populations of children with APD and their mothers (limited to those who have participated in auditory therapy), and the number of participants is relatively small. We also cannot be certain that the observed changes in the relationships between stress and personality traits in mothers with APD children, and in those mothers with TD children, were due to the COVID-19 pandemic. Other coexisting factors—such as a fear of contracting COVID-19, the loss of family members due to the disease, and the availability of social support during the pandemic—could have been different for mothers with APD and mothers of TD children.

## 5. Conclusions

The COVID-19 pandemic can be regarded as a time that brought about changes in the psychological functioning of mothers with TD children, although, curiously, not in mothers with APD children. The evidence can be seen in measures of the levels of personality traits, especially emotional stability, which underwent changes in the later phase of the pandemic. Both groups of mothers showed somewhat different relationships between personality traits and self-perceived global stress during the later phase than before the pandemic. Emotional stability was a particularly sensitive indicator, and here we detected a significant interaction between it and the factor of APD vs. TD child, as well as between it and the factor of pandemic phase (pre- vs. late). Somewhat paradoxically, perhaps, we observed that mothers with TD children had a greater risk of poorer psychological functioning (as indicated by a significant deterioration in emotional stability late in the pandemic) than mothers with APD children. Mothers with reduced well-being (including those with low emotional stability in particular) were at risk of higher global stress, which might also have had consequences for their children’s psychological functioning. Children of these mothers were more likely to be emotionally or even physically abused or neglected, and here, psychological support is important. Appropriate parent- and family-focused psychological treatments might include counseling, psychotherapy, or other types of psychological intervention, as well as parent/family support groups. Special psychological care and support should also be aimed at children of parents with poor mental health. 

## Figures and Tables

**Table 1:** Sociodemographic data on the mothers of children with APD, mothers of TD children, and the children themselves collected during the later part of the COVID-19 pandemic.

	Mothers of APD Children	Mothers of TD Children	Significance of Intergroup Comparison
*N*	108	79	
Age (years)—M (SD)	40.79 (4.82)	41.3 (5.18)	*t* = −0.688; *p* = 0.492
Education			
Primary and secondary—*n* (%)	78 (72.8)	70 (88.6)	*χ*^2^ = 7.42; *p* = 0.0064 ^a^
Tertiary—*n* (%)	30 (27.8)	9 (11.4)	
Marital/partnership status			
in a relationship—*n* (%)	89 (82.4)	64 (85.3)	*χ*^2^ = 0.28; *p* = 0.599
single—*n* (%)	19 (17.6)	11 (14.7)	
Number of children—M (SD)	2.3 (0.87)	2.0 (0.81)	*Z* = 1.632; *p* = 0.103
one—*n* (%)	18 (16.7)	23 (29.1)	*χ*^2^ = 6.59; *p* = 0.089
two—*n* (%)	55 (50.9)	34 (43.0)	
three—*n* (%)	25 (23.1)	20 (25.3)	
four—*n* (%)	9 (8.3)	2 (2.5)	
five—*n* (%)	1 (0.9)	0 (0)	
	Children with APD	TD children	
*N*	108	79	
Sex			
Girls—*n* (%)	43 (39.8)	38 (48.1)	*χ*^2^ = 1.28; *p* = 0.26
Boys—*n* (%)	65 (60.2)	41 (51.9)	
Age (months)—M (SD)	125.9 (22.1)	123.9 (32.9)	*t* = 0.51; *p* = 0.616
	Min = 84	Min = 71	
	Max = 192	Max = 198	
Education			
Preschool education (6 y.o.)—*n* (%)	0 (0)	5 (6.3)	*χ*^2^ = 11.99; *p* = 0.0074 ^a^
Early primary education (7–9 y.o.)—*n* (%)	26 (24.1)	26 (32.9)	
Primary education (10–14 y.o.)—*n* (%)	78 (72.2)	42 (53.2)	
Secondary education (15–18 y.o.)—*n* (%)	4 (3.7)	6 (7.6)	

**^a^** Significant difference.

**Table 2:** Effects of the variables Group (APD vs. TD, first column) and COVID-19 stage (pre- vs. late, second column), as well as the interaction between the two (third column), on self-perceived stress level (PSS-10) and personality characteristics (IPIP-BFM-20). Four groups of subjects are included: two as listed in Table 1 and two from Table 1 of [13]. The means for the presented parameters are shown in Table 3 and Table 4 of this study and Table 2 of [13]. *F*—test statistics; *p*—probability level.

Variable	Group; *F* (*p*)	COVID-19 Pandemic Stage; *F* (*p*)	Interaction; *F* (*p*)
Stress level	0.66 (0.417)	0.724 (0.396)	0.024 (0.876)
Extraversion	1.976 (0.161)	0.041 (0.839)	0.065 (0.799)
Agreeableness	4.192 (0.041) ^a^	0.816 (0.367)	0.003 (0.956)
Conscientiousness	2.708 (0.101)	1.27 (0.26)	1.687 (0.195)
Emotional stability	1.674 (0.197)	0.74 (0.39)	5.239 (0.023) ^a^
Intellect/Imagination	14.157 (<0.001) ^a^	1.455 (0.229)	2.106 (0.148)

^a^ Significant difference.

**Table 3:** Comparison of self-perceived stress level (PSS-10) and personality characteristics (IPIP-BFM-20) between groups of mothers of APD children before the COVID-19 pandemic (Table 2 of [13]) and late in the COVID-19 pandemic.

Variable	Mothers of APD Children before COVID-19; M (SD)	Mothers of APD Children Late in COVID-19; M (SD)	*t*	*p*
Self-perceived stress level	17.69 (6.67)	17.24 (6.21)	0.517	0.605
Extraversion	11.09 (3.57)	11.07 (3.26)	0.040	0.968
Agreeableness	13.83 (2.59)	13.57 (2.58)	0.731	0.466
Conscientiousness	12.63 (3.29)	12.69 (2.86)	–0.133	0.895
Emotional stability	9.06 (2.97)	9.52 (2.9)	–1.135	0.258
Intellect/Imagination	11.9 (2.79)	12.63 (2.74)	–1.943	0.053

**Table 4:** Comparison of self-perceived stress level (PSS-10) and personality characteristics (IPIP-BFM-20) between groups of mothers of TD children before the COVID-19 pandemic (Table 2 of [13]) and late in the COVID-19 pandemic.

Variable	Mothers of TD Children before COVID; M (SD)	Mothers of TD Children during COVID; M (SD)	*t*	*p*
Self-perceived stress level	17.27 (4.96)	16.61 (6.81)	0.694	0.488
Extraversion	11.51 (3.48)	11.67 (3.43)	–0.299	0.765
Agreeableness	14.37 (2.43)	14.14 (2.63)	0.565	0.573
Conscientiousness	13.58 (3.21)	12.8 (2.99)	1.589	0.114
Emotional stability	10.2 (2.92)	9.2 (3.38)	1.989	0.048 ^a^
Intellect/Imagination	13.33 (2.5)	13.27 (2.34)	0.175	0.861

M—mean; SD—standard deviation; ^a^ significant difference.

**Table 5:** Pearson correlation coefficients between self-perceived stress (PSS-10) and personality characteristics (IPIP-BFM-20) of mothers of APD children and mothers of TD children late in the COVID-19 pandemic.

	Extraversion	Agreeableness	Conscientious-ness	Emotional Stability	Intellect/Imagination
APD					
Self-perceived stress	–0.2802 (*p* = 0.003) ^a^	–0.2351 (*p* = 0.014) ^a^	–0.1675 (*p* = 0.083)	–0.5573 (*p* < 0.001) ^a^	–0.3315 (*p* < 0.001) ^a^
TD					
Self-perceived stress	–0.2868 (*p* = 0.010) ^a^	–0.0519 (*p* = 0.650)	–0.0448 (*p* = 0.695)	–0.6022 (*p* < 0.001) ^a^	–0.0570 (*p* = 0.618)

^a^ Significant correlations.

**Table 6:** Regression model of predictors of self-perceived stress level (PSS-10) of mothers of APD children and mothers of TD children late in the COVID-19 pandemic. The dependent variable is self-perceived stress.

	*β*	SE *β*	*B*	SE *B*	*t*	*p*
APD						
Extraversion	−0.11833	0.1052	–0.3646	0.2092	–1.7427	0.0856
Agreeableness	0.0725	0.1027	0.1875	0.2655	0.7064	0.4822
Conscientiousness	−0.0248	0.0941	–0.0564	0.2142	–0.2635	0.7929
Emotional stability	−0.5688	0.0955	–1.1455	0.1924	–5.9547	<0.0001 ^a^
Intellect/Imagination	0.0562	0.1028	0.1639	0.2999	0.5465	0.5864
TD						
Extraversion	−0.1361	0.0876	–0.2595	0.1669	–1.5543	0.1232
Agreeableness	−0.0786	0.0828	–0.1891	0.1992	–0.9495	0.3446
Conscientiousness	−0.1843	0.0768	–0.4004	0.1668	–2.4005	0.0182 ^a^
Emotional stability	−0.5015	0.0798	–1.0718	0.1705	–6.2853	<0.0001 ^a^
Intellect/Imagination	−0.1126	0.0879	–0.2551	0.1992	–1.2807	0.2032

^a^ Significant effects.

## Data Availability

Data will be shared when contacting the corresponding author upon reasonable request.
